# Multidimensional optimization for accelerating light-powered biocatalysis in* Rhodopseudomonas palustris*

**DOI:** 10.1186/s13068-023-02410-3

**Published:** 2023-10-27

**Authors:** Yang Zhang, Wenchang Meng, Yuting He, Yuhui Chen, Mingyu Shao, Jifeng Yuan

**Affiliations:** 1 State Key Laboratory of Cellular Stress Biology, School of Life Sciences, Faculty of Medicine and Life Sciences, Xiamen University, Fujian, 361102 China; 2 Shenzhen Research Institute of Xiamen University, Shenzhen, 518057 China

**Keywords:** Light-driven biocatalysis, Cofactor regeneration, Lignocellulose upcycling, Isoprenol utilization pathway, *Rhodopseudomonas palustris*

## Abstract

**Background:**

Whole-cell biocatalysis has been exploited to convert a variety of substrates into high-value bulk or chiral fine chemicals. However, the traditional whole-cell biocatalysis typically utilizes the heterotrophic microbes as the biocatalyst, which requires carbohydrates to power the cofactor (ATP, NAD (P)H) regeneration.

**Results:**

In this study, we sought to harness purple non-sulfur photosynthetic bacterium (PNSB) as the biocatalyst to achieve light-driven cofactor regeneration for cascade biocatalysis. We substantially improved the performance of *Rhodopseudomonas palustris*-based biocatalysis using a highly active and conditional expression system, blocking the side-reactions, controlling the feeding strategy, and attenuating the light shading effect. Under light-anaerobic conditions, we found that 50 mM ferulic acid could be completely converted to vanillyl alcohol using the recombinant strain with 100% efficiency, and > 99.9% conversion of 50 mM *p*-coumaric acid to *p*-hydroxybenzyl alcohol was similarly achieved. Moreover, we examined the isoprenol utilization pathway for pinene synthesis and 92% conversion of 30 mM isoprenol to pinene was obtained.

**Conclusions:**

Taken together, these results suggested that *R. palustris* could be a promising host for light-powered biotransformation, which offers an efficient approach for synthesizing value-added chemicals in a green and sustainable manner.

**Graphical Abstract:**

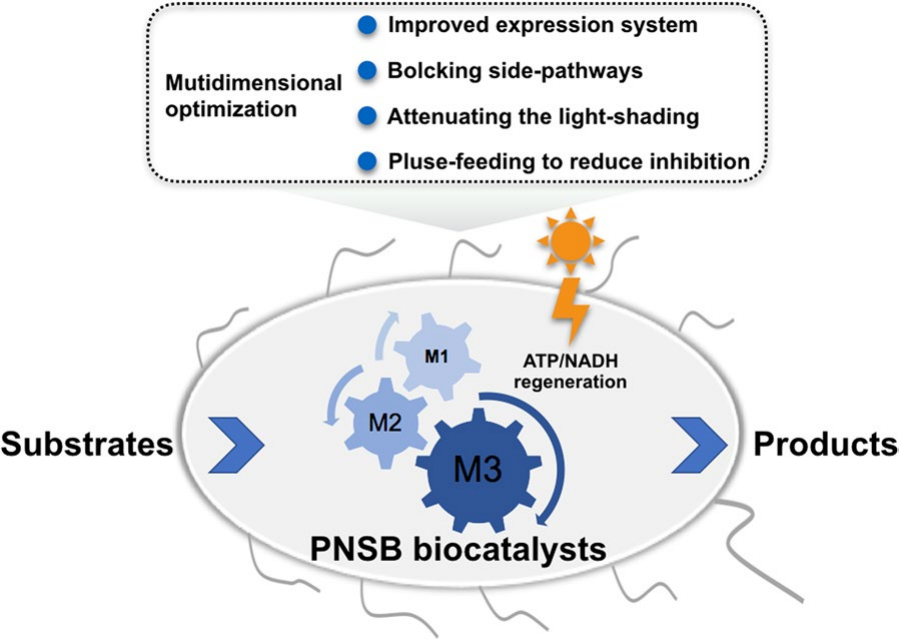

**Supplementary Information:**

The online version contains supplementary material available at 10.1186/s13068-023-02410-3.

## Background

Biocatalysis plays an essential part of facilitating chemical synthesis in a greener way, as enzymes are usually highly active and operate under mild conditions [1, 2]. The diversity of nature provides a large number of multistep catalytic reactions under the most benign conditions that can be utilized for the production of a variety of chemicals. With the development of synthetic biology, exploiting the enzyme cascades for multistep conversions of renewable feedstocks could afford the relatively toilless admittance to various types of useful value-added compounds [3, 4]. Because of these fascinating features and the dramatic increase in practicable enzymes, cascade enzymatic conversion is turning into a rapid expanding field for chemical synthesis [5].

Different types of catalysts, such as purified enzymes, immobilized enzymes, cell-free extracts, whole cells, or a mixture of them, can be applied in cascade biocatalysis. A specially-made whole-cell biocatalyst harboring all needed enzymes is regarded as the most economical option among these forms, owing to the advantages as follows [6, 7]: (1) cells are harvested through cultivation at relatively low cost without further downstream purification process; (2) intracellular environment offers cofactor generation to support the reactions; (3) enzymes could be protected with increased life span by cell walls and membranes; (4) co-expression of multiple enzymes within the cells enhances the local concentrations of required enzymes and subsequently decreases the intermediate diffusion in multistep reactions.

In recent years, whole-cell biotransformation has been exploited to convert a variety of substrates such as alkanes, phenols, styrene, fatty acids, ketones, amino acids, and terpene derivatives, into high-value bulk or chiral fine chemicals [7]. The heterotrophic microorganism of *Escherichia coli* represents the most-widely used whole-cell biocatalytic system, and other heterotrophic microorganisms such as *Corynebacterium glutamicum* [8], *Bacillus subtilis* [9], and *Pseudomonas putida* [10] are being established in the recent years. However, all these heterotrophic microorganisms need carbohydrates to support the biomass accumulation and cofactor regeneration, which pose additional costs for their industrial development. For instance, to supply additional ATP and NAD(P)H during the biocatalytic processes [11–14], additional glucose is added for cofactor regeneration through endogenous cell metabolisms, and in many cases, glucose dehydrogenase (GDH) was supplemented for enhancing NAD(P)H regeneration [13, 15].

Purple non-sulfur photosynthetic bacterium (PNSB) is widely distributed in nature and it shows extraordinarily versatile metabolism such as photoautotrophic mode using carbon from CO_2_, and energy from light and inorganic electron donors [16]. In particular, the photosynthetic system absorbs light energy to drive electrons transfer along with the generation of a proton gradient across the membrane. Using energy stored in the proton gradient, ATP synthase and NADH-quinone oxidoreductase catalyze the formation of ATP and NADH that are essential for CO_2_ fixation and other metabolic reactions [17]. The ability of utilizing CO_2_ (greenhouse gas) as the carbon source and light energy to support the cell growth and cofactor regeneration makes PNSB a promising and economic whole-cell biocatalyst.

**Fig. 1:**
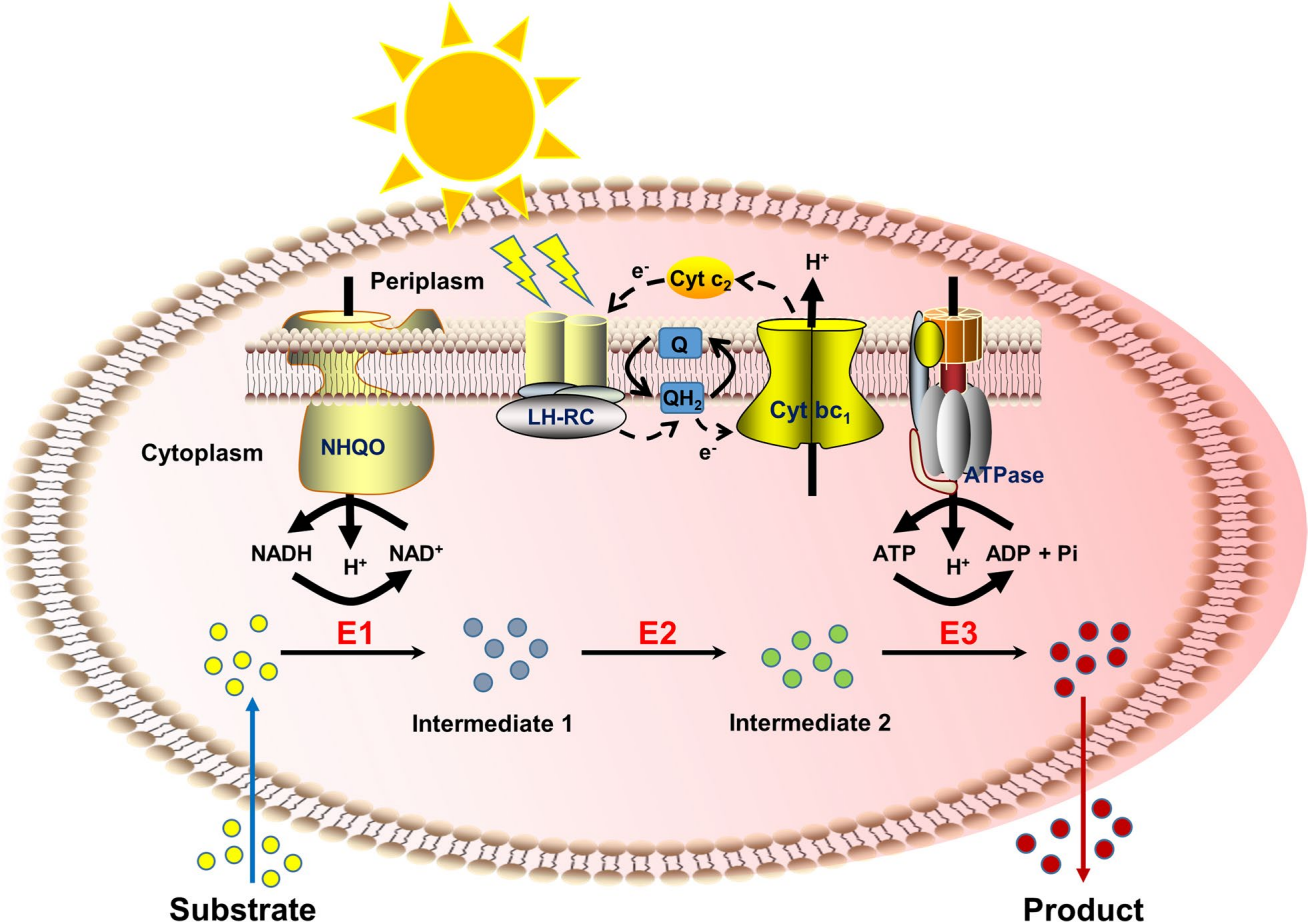
Schematic diagram of light-driven cofactor regeneration for biotransformation using PNSB. Upon excitation by the absorption of light or by energy transfer from light-harvesting complexes, the photosynthetic reaction center reduces ubiquinone (Q) to ubiquinol (QH_2_). Cytochrome *bc*_1_ complexes (Cyt *bc*_1_) oxidize QH_2_ and transfer protons (H^+^) across the membrane generating the proton motive force. As a result, ATP and NADH are, respectively, produced by F_0_F_1_-ATP synthase (ATPase) and NADH: quinone oxidoreductase (NHQO). The dotted arrow shows the path of cyclic electron transfer chain from the photosynthetic reaction center to ubiquinone, to Cyt *bc*_1_, and to soluble Cyt *c*_2_. The enzymes (E1–E3) catalyze the reactions wherein the substrate gets transformed to the product with an expense of the cofactors ATP and NADH from anoxygenic photosynthesis. LH-RC: light harvesting complexes and photosynthetic reaction center

In this study, we attempted to develop *Rhodopseudomonas palustris* as a platform for light-driven cofactor regeneration to power the biocatalytic processes (Fig. 1). Upon multilevel optimization of the protein expression system, blocking the side-reactions, controlling the feeding strategy and attenuating the light shading effect, we managed to substantially improve the performance of PNSB-based biocatalysis. We found that vanillyl alcohol (VA) known as a flavoring agent, *p*-hydroxybenzyl alcohol (*p*HBA) known as an organic synthetic intermediate, and pinene known as a precursor to biofuels were produced near 100% conversion with light-driven cofactor regeneration. Taken together, our work lays a new foundation to harness PNSB as the biocatalyst for chemical productions.

## Results

### Development of low-oxygen induced protein expression system under anaerobic conditions

**Fig. 2:**
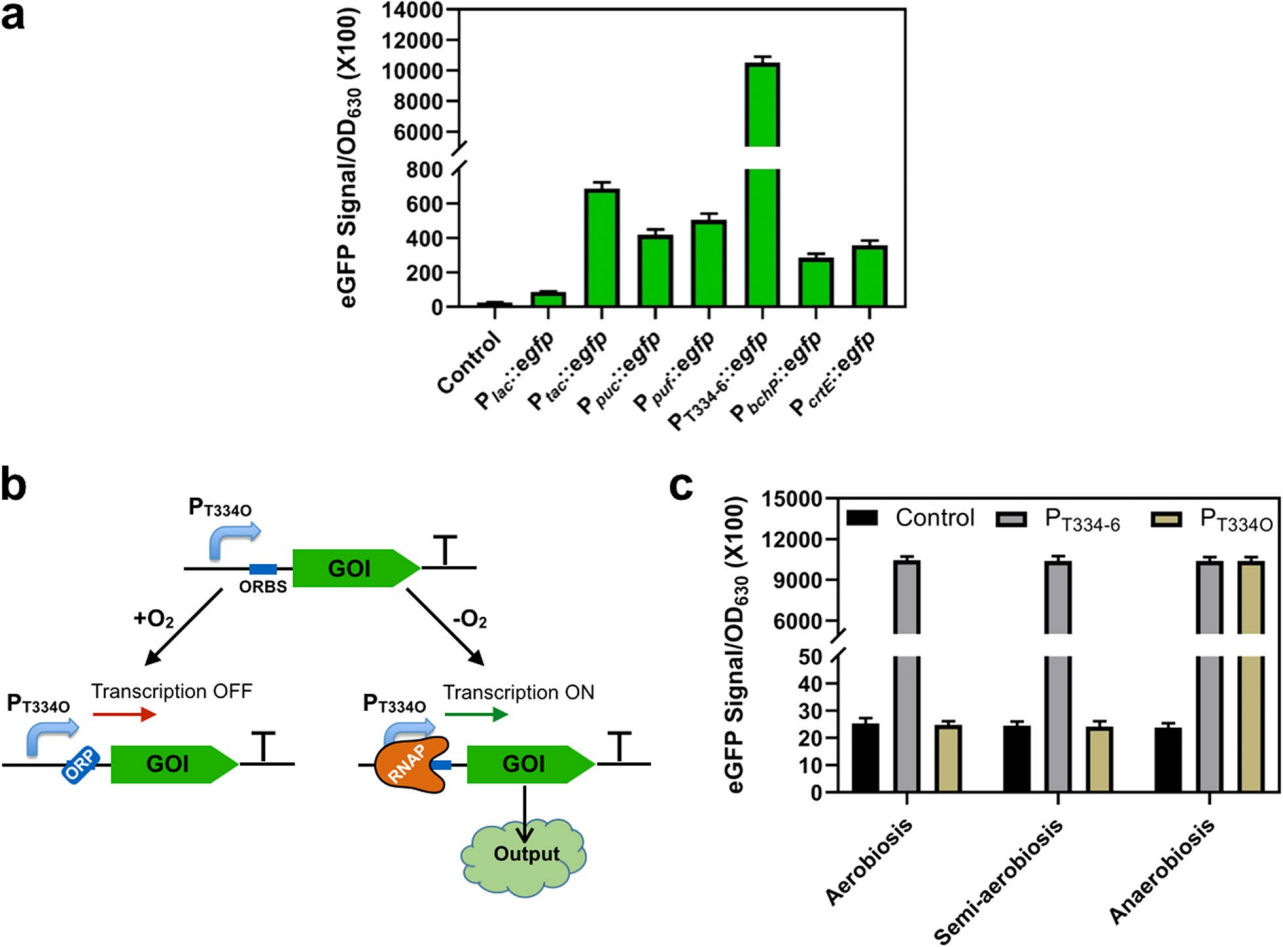
Development of low-oxygen induced protein expression system in *R. palustris*. **a** The promoter activities under light-anaerobic conditions were compared by eGFP fluorescence intensities. The synthetic promoter of P_T334O_, the heterologous promoters of P_*lac*_ and P_*tac*_ from *E. coli*, and the endogenous promoters of P_*puc*_, P_*puf*_, P_*bchP*_ and P_*crtE*_ were analyzed. **b** Schematic design of low-oxygen induced protein expression based on the synthetic promoter P_T334O_. ORP: oxygen regular proteins, ORBS: oxygen regular protein binding site, RNAP: RNA polymerase, GOI: genes of interest. **c** The activities of P_T334-6_ and P_T334O_ were tested at different oxygen levels. The control indicates the strain with the empty plasmid pBRT. All experiments were carried out in triplicate, and the data are presented as mean and standard deviations

Since the abundance of enzymes is crucial for efficient whole-cell biotransformation, there is a pressing need to develop a highly-active protein expression system in *R. palustris.* In this study, several promoters were employed to drive the reporter gene of *egfp* in *R. palustris*, including promoters of *puc* operon (P_*puc*_), *puf* operon (P_*puf*_), *bch* operon (P_*bchP*_) and *crt* operon (P_*crtE*_) from *R. palustris*, P_T334-6_ from *Rhodobacter sphaeroides*, *lac* operon (P_*lac*_) and its variant P_*tac*_ from *E. coli*. Specifically, the expression levels of *puc* and *puf* operon encoding the light-harvesting complex II and complex I are relatively high under light-anaerobic conditions; *bch* operon takes charge of the bacteriochlorophyll synthesis and *crt* operon is responsible for the carotenoid synthesis; P_T334-6_, a P_*rsp_7571*_-derived synthetic promoter, showed 32-fold higher activity than that of P_*tac*_ under the testing condition in *R. sphaeroides* [18]. As depicted in Fig. 2a, five endogenous promoters displayed much higher activity than P_*lac*_ but lower than P_*tac*_, and the synthetic promoter of P_T334-6_ exhibited approximately 14-fold higher activity than that of P_*tac*_ under light-anaerobic conditions.

Ideally, the expression of enzymes should be repressed during chemoheterotrophic growth under aerobic conditions, to prevent triggering any protein burden and remain stable maintenance of the genetic construct. According to a previous report, a hybrid promoter of *lacI*^q^-P_*puc*_ was constructed, which is tightly regulated by both isopropyl *β*-D-thiogalactoside (IPTG) and low oxygen level [19]. Due to lack of oxygen-regulatory proteins (ORP) binding site information of endogenous P_*puc*_ from *R. palustris*, the ORP binding site of P_*puc*_ from *R. sphaeroides* [20] was chosen to insert at the downstream of P_T334-6_ to design P_T334O_ with the aim of using oxygen as the inhibitor (Fig. 2b). As shown in Fig. 2c, P_T334-6_ activity did not show obvious change at different oxygen levels, indicating that P_T334-6_ itself is insensitive to oxygen tension. Excitingly, we found that ORP of *R. palustris* could interact with ORP binding site from *R. sphaeroides*. As shown in Fig. 2c, P_T334O_ exhibited a comparable activity to P_T334-6_ under anaerobic conditions suggesting that the synthetic hybrid promoter of P_T334O_ is tightly activated by low oxygen tension, while P_T334O_ had no obvious activity under aerobic conditions or semi-aerobic conditions. Therefore, the synthetic hybrid promoter of P_T334O_ was used for the subsequent cascade biotransformation in *R. palustris*.

### Synthesis of vanillyl alcohol from ferulic acid by cascade biotransformation

Upon the construction of a highly active and controllable protein expression system, we next proceeded to examine PNSB as the biocatalyst for chemical productions. Lignocellulose is the most abundant renewable biomass on earth that consists of cellulose (30–35%), hemicellulose (25–30%), and lignin (10–25%). Ferulic acid (FA) is one of the common aromatic monomers obtained from hemicellulose and lignin depolymerization through various alkaline, acidic and enzymatic methods [21]. It is well known that *R. palustris* has a robust metabolism to degrade a variety of aromatic compounds, and thus it has a great potential to be a platform for synthesis of high value-added aromatic compounds from renewable lignin-based substrates. To demonstrate the capability of light-driven cofactor regeneration for improved biocatalysis, we proceeded to investigate the biotransformation of FA into vanillyl alcohol (VA), a natural aromatic compound found in several plants such as *Gastrodia elata* Blume and *Vanilla planifolia*, which is known to exhibit a variety of pharmacological activities including antioxidant, anti-inflammatory, anti-nociceptive, anti-asthmatic, and anti-convulsive activities [22, 23].

**Fig. 3:**
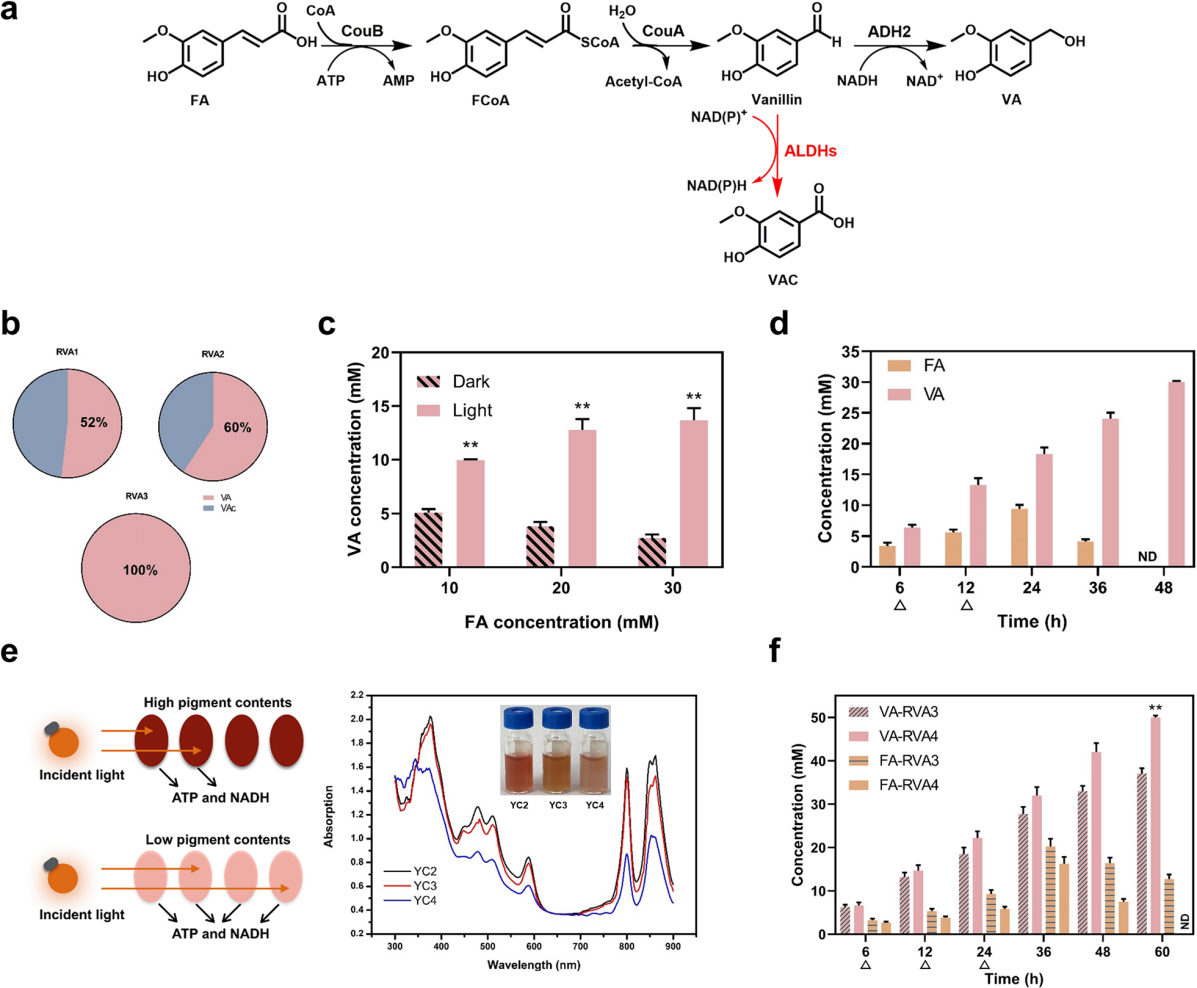
Synthesis of VA from FA by *R. palustris* mediated whole-cell biocatalysis. **a** Schematic diagram of the biocatalytic route for VA synthesis from FA. FA: ferulic acid, FCoA: feruloyl-CoA, VA: vanillyl alcohol, VAC: vanillic acid. **b** The distribution profile of the products from 5 mM FA using different recombinant strains. Strains RVA1, RVA2, and RVA3 represent the base strain, the mutant with *RPA1206* deletion, and the mutant with *RPA1206*, *RPA1687* and *RPA1725* deletions, respectively, containing CouBA-ADH2.** c** The effect of light exposure on the VA productions from different concentrations of FA incubating with the resting cells of RVA3 under dark- and light-anaerobic conditions. Two asterisk indicates statistically significant results (*p* < 0.005). **d** Time course of VA production from 30 mM FA by RVA3 using the pulse-feeding approach with supplementing 10 mM FA every 6 h under light-anaerobic conditions. The triangles represent for the feeding points. **e** Manipulation of carotenoid synthesis for reduced light shading. The absorption spectra and colour appearance of *R. palustris* under light-anaerobic conditions are provided. Strain YC3 (ispA^*^), and YC4 (ispA^*^crtE^*^) represent the restriction of *ispA* and both *ispA* and *crtE*, respectively. **f** VA synthesis from 50 mM FA using strain RVA3 and RVA4 under the pulse-feeding mode by periodically adding 10 mM FA every 6 h. The triangles represent for the feeding points. All experiments were conducted in triplicate. Data represent the mean and standard deviations

As shown in Fig. 3a, the enzymatic cascade contains: FA condenses with coenzyme A (CoA) with expense of ATP by the CoA ligase (encoded by *couB* from *R. palustris*), then feruloyl-CoA undergoes hydration and cleavage to vanillin with the enoyl-CoA hydratase/aldolase (encoded by *couA* from *R. palustris*) [24], and further reduction of vanillin by utilizing the NADH dependent alcohol dehydrogenase encoded by *adh2* from *Saccharomyces cerevisiae* [25] to form VA. We, therefore, constructed two plasmids with modular expression of *couBA* from *R. palustris* and *adh2* from *S. cerevisiae* (Additional file 1: Fig. S1). Given that CouB and ADH2 require the presence of respective cofactors of ATP and NADH, this biocatalytic route is an excellent choice for investigating the effect of cofactor supply. As depicted in Fig. 3b, 2.6 mM VA (52%) and 2.4 mM vanillic acid (VAC, 48%) were produced from 5 mM FA using strain RVA1 (the base strain of *R. palustris* harboring the enzyme cascade of CouBA-ADH2). These findings suggested that PNSB could efficiently intake the substrate and convert FA into vanillin-derived products. However, the accumulation of VAC suggested that the endogenous aldehyde dehydrogenases (ALDHs) could be a limiting factor to achieve efficient VA production in *R. palustris*.

### Identifying the endogenous factors limiting the vanillyl alcohol synthesis

To avoid the formation of VAC byproduct, we first deleted *RPA1206* (Additional file 1: Fig. S2a), encoding an ALDH with reported activity on oxidizing *p-*hydroxybenzylaldehyde to *p*-hydroxybenzoic acid under anaerobic conditions [26]. However, the proportion of VA in strain RVA2 (strain YC1 harboring CouBA-ADH2) was slightly improved from 52 to 60%, suggesting that there are other ALDHs involved in VAC formation. Next, we proceeded to delete *RPA1687* (coniferyl aldehyde reductase) and *RPA1725* (aldehyde reductase to phenolic acid) to further disrupt VAC formation from vanillin (Fig. S2a). Encouragingly, 5 mM VA with ~ 100% conversion was obtained in *R. palustris* RVA3 (strain YC2 harboring CouBA-ADH2) and no VAC was detected (Fig. 3b and S2d). In addition, there was no obvious differences of cell growth and light absorption between the base strain and the mutant strain with triple deletion of *RPA1206*, *RPA1687* and *RPA1725* (Additional file 1: Fig. S2b and S2c).

To confirm the essentiality of light to power the biocatalysis, we next compared the VA production between dark- and light-anaerobic conditions. As depicted in Fig. 3c, we found that the conversions of FA to VA were much higher under light-anaerobic conditions when compared to those under dark conditions. For instance, 13.7 mM (211.2 mg/g-cdw) were obtained with 45.7% conversion under light-anaerobic conditions, whereas only 2.7 mM (41.6 mg/g-cdw) with 9.1% conversion were reached under dark-anaerobic conditions. Therefore, we concluded that light plays an indispensable role in regenerating the cofactors to accelerate the biotransformation. Since the biocatalytic system experienced a severe substrate inhibition, we next proceeded the pulse-feeding strategy by periodically adding 10 mM FA every 6 h and 30 mM FA was fully converted into VA within 48 h (Fig. 3d).

### Attenuating the light shading effect for improved biocatalytic efficiency

Since whole-cell biotransformation typically uses high-cell density for the catalysis, the pigment content in *R. palustris* would cause the shading effect that prevents the transmission ability of light, thereby diminishes the cofactor regeneration (Fig. 3e). In *R. palustris*, the main pigment is attributed to the carotenoid content, which is synthesized from the methylerythritol phosphate (MEP) pathway. Given farnesyl pyrophosphate synthase (FPPS encoded by *ispA*) and geranylgeranyl diphosphate synthase (GGPPS encoded by *crtE*) are involved in carotenoids synthesis, the promoter P_*lac*_ with a relatively low activity (Fig. 2b) was used to replace the promoters of *ispA* and *crtE* to obtain *R. palustris* YC3 (ispA^*^) and YC4 (ispA^*^crtE^*^) based on the control stain YC2 as shown in Additional file 1: Figure S3a. The decrease of *ispA* expression resulted in a slightly lower light absorption compared to that of the control due to the presence of *crtE* while the decreases of *ispA-crtE* expressions gave rise to the remarkably lower light absorption (Fig. 3e), implying that the pigment biosynthesis was much weakened. Besides, the colour appearance of strains correlated well with the light absorption profile (Fig. 3e). Notably, we did not observe noticeable change of the growth of all the recombinant strains (Additional file 1: Fig. S3b), suggesting that the above-mentioned modifications did not damage the photosynthetic ability.

When the engineered strains with less light shading were used for the biocatalytic formation of VA from FA, the overall catalytic efficiencies were substantially improved in these strains. As shown in Fig. 3f, strain RVA4 (YC4 harboring CouBA-ADH2) displayed a better performance at high FA concentration, and 50 mM FA was converted to 770.8 mg/g-cdw VA with > 99.9% conversion within 60 h, whereas only 74.1% conversion was obtained by the control of RVA3. Therefore, we have further improved the performance of PNSB-mediated biocatalyst by attenuating the light shading, which would be of significant interest for the future industrial applications when the light transmission becomes a bottleneck.

### Synthesis of *p*-hydroxybenzyl alcohol from *p*-coumaric acid by cascade biotransformation

To demonstrate the broad utility of CouBA-ADH2, we also used the same enzyme cascade to produce *p*-hydroxybenzyl alcohol (*p*HBA) from *p*-coumaric acid (*p*CA), another abundant lignocellulose-derived compound (Additional file 1: Fig. S4a). When *p*CA concentration increased from 5 to 30 mM, 5.0 to 15.1 mM (62.1 to 187.5 mg/g-cdw) *p*HBA were gained by RHBA1 (the same as the strain RVA3) along with substrate conversion descending from 100% to 50.3% under light-anaerobic conditions, whereas low titers of 3.5 to 3.2 mM (43.4 to 39.7 mg/g-cdw) *p*HBA along with 70% to 10.7% conversion were synthesized under dark conditions as described in Additional file 1: Figure S4b. Moreover, no *p*-hydroxybenzoic acid (*p*HBAC) was observed during the biocatalysis based on the HPLC result (Additional file 1: Fig. S5). We further proceeded the pulse-feeding strategy to reduce substrate inhibition and 371.9 mg/g-cdw *p*HBA with about 100% conversion from 30 mM *p*CA was achieved in 48 h (Additional file 1: Fig. S4c). In addition, we also examined the engineered strain with attenuated photosynthetic pigments and more efficient *p*HBA synthesis was obtained in strain RHBA2 (the same as the strain RVA4) at high* p*CA concentration of 50 mM. Specially, the substrate of *p*CA was fully transformed into* p*HBA of 620.7 mg/g-cdw within 60 h, while only 76.2% conversion was reached by the control and 10.9 mM substrate remained (Additional file 1: Fig. S4d). Therefore, we concluded that the enzyme cascade of CouBA-ADH2 could be applied for synthesizing other aromatic alcohols by simply varying lignocellulose-derived monomers.

### Synthesis of pinene from isoprenol by cascade biotransformation

**Fig. 4:**
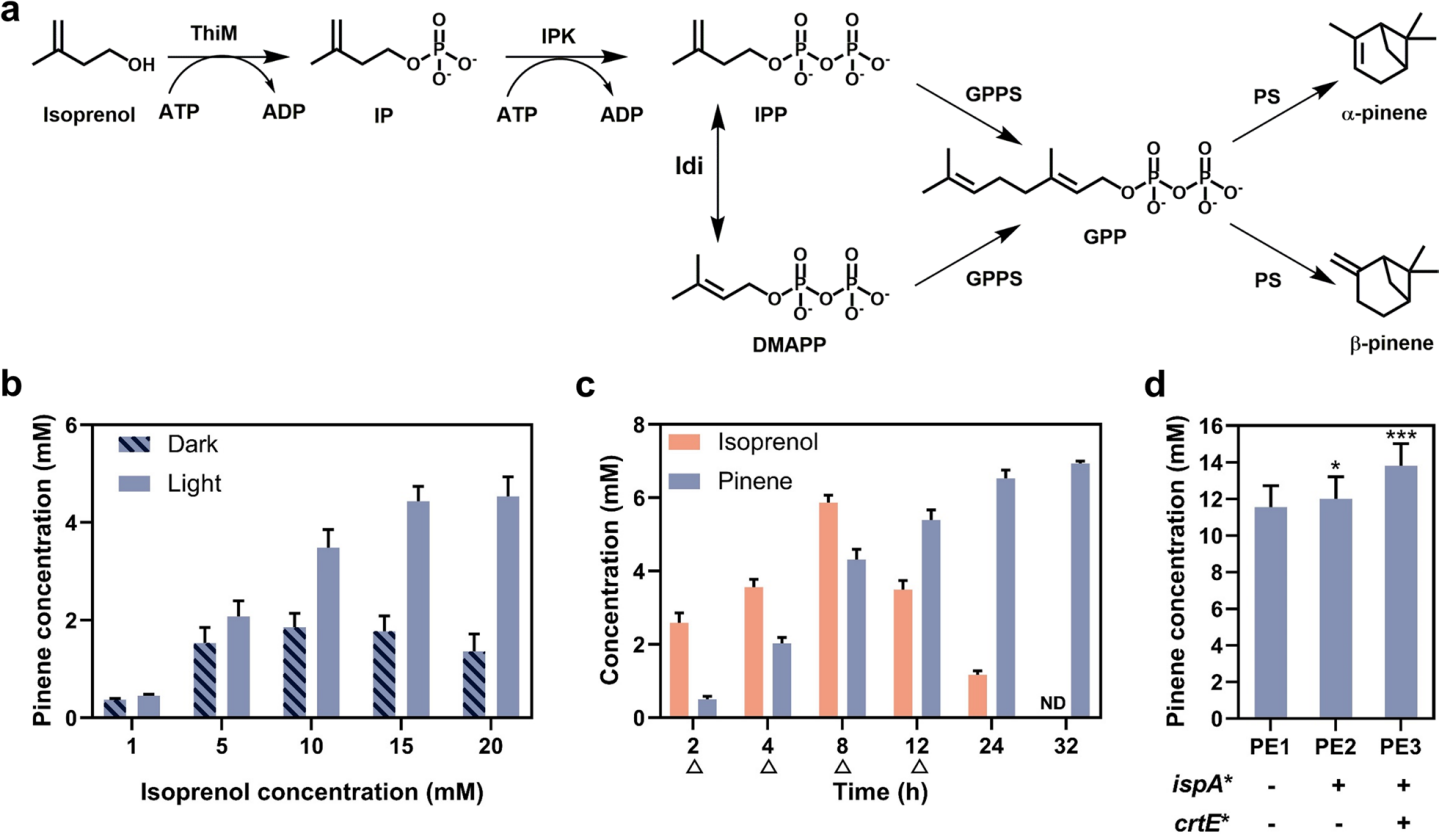
Synthesis of pinene from isoprenol by *R. palustris* mediated whole-cell biocatalysis*.* **a** Schematic diagram of IUP-coupled pinene synthesis from isoprenol. **b** Biotransformation of isoprenol into pinene under dark- and light-anaerobic conditions by strain PE1. **c** Time course of the pinene production from 20 mM isoprenol by strain PE1 using the pulse-feeding approach under light-anaerobic conditions. The triangles represent for the feeding points. **d** Biotransformation of 30 mM isoprenol into pinene by the engineered strains with reduced light shading using the pulse-feeding approach under light-anaerobic conditions. Strain PE1, PE2, and PE3 represent the base strain, YC5 and YC6 harboring the cascades ThiM-IPK-Idi-GPPSps, respectively. All experiments were conducted in triplicate. Data represent the mean and standard deviations. One asterisk indicates statistically significant results (*p* < 0.05) and three asterisks indicate *p* < 0.001

To further expand the applicability of light-powered biocatalysis, we also examined isoprenol utilization pathway (IUP) based terpene synthesis. In this study, we used pinene synthesis from isoprenol as an example, which requires large amounts of ATP for substrate phosphorylation (Fig. 4a). Cascade 1: isoprenol undergoes two-step phosphorylation to isopentenyl diphosphate (IPP) using an *E. coli* kinase (ThiM) [27] and isopentenyl phosphate kinase (IPK) from *Arabidopsis thaliana* [28], followed by interconversion of IPP and dimethylallyl diphosphate (DMAPP) using isopentenyl pyrophosphate isomerase (Idi) from *E. coli*. Cascade 2: condensation of IPP and DMAPP by geranyl diphosphate synthase (GPPS) from *Abies grandis* gives GPP which serves as the substrate for pinene synthesis via the further action by pinene synthase (PS) from *A. grandis* [29]. To mitigate the competing pathways of host metabolism, we used a direct fusion of GGPS with PS as previously reported [29].

The *R. palustris* containing the desired enzyme cascades (Additional file 1: Fig. S6) was then tested for pinene synthesis from isoprenol in a two-phase system containing 20% *n*-dodecane. As depicted in Fig. 4b, the yields of pinene under light-anaerobic conditions reached 6.1–61.8 mg/g-cdw with 90–45.4% conversion, which were much higher than those of 5.0–18.5 mg/g-cdw with 74–13.6% conversion under dark conditions. These results suggested that the light-driven ATP regeneration in *R. palustris* is still effective in improving the overall conversion of isoprenol to pinene. Since the fusion proteins of GPPS-PS could create a direct channel between GPP and pinene that facilitates the flux to product (pinene) and alleviates the GPP inhibition to PS [30], and two-phase system could alleviate the pinene toxicity and its inhibition to PS, we reasoned that the low titers of pinene biosynthesis might be caused by the toxicity of isoprenol to the host or substrate inhibition of isoprenol. We next proceeded with periodical addition of 4 mM isoprenol every 2 h until the final content reached 20 mM by utilizing pulse-feeding strategy. We were excited to find that 6.9 mM pinene was achieved and the conversion was substantially enhanced from 45% of direct feeding to 69% (Fig. 4c), and no isoprenol was detected after 32 h (Additional file 1: Fig. S7). Furthermore, the engineered strains with further restriction the metabolic flux towards by-products of carotenoids and reduced light-shading were also examined for pinene synthesis from 30 mM isoprenol. As shown in Fig. 4d, we found the engineered strain with reduced light shading and restriction the metabolic flux towards by-products of carotenoids could substantially improve the overall conversion of isoprenol to pinene, reaching 13.8 mM pinene (188 mg/g-cdw) in strain PE3 (YC6 derivative) with 92% conversion.

## Discussion

Biocatalysis is considered to be a sustainable and environment-friendly alternative to chemical synthesis. Although cyanobacterium affords encouraging source of reducing power of NADPH [31, 32], it is not ideal for NADH regeneration. Given its adaptable metabolism and ability to catabolize a wide variety of feedstocks such as hexose, pentose, volatile organic acid, aromatics, and etc., *R. palustris* has been studied and applied in biodegradation of aromatic compounds, environmental remediation, biofuel production, agricultural biostimulation, and bioelectricity production [16]. In this study, we have developed *R. palustris* as the whole-cell biocatalyst, and harnessed the light to power cofactor regeneration (ATP and NADH) for biotransformation applications. The performance of PNSB-based biocatalysis was substantially improved using a highly active and conditional expression system, blocking the side-reactions, controlling the feeding strategy and attenuating the light shading effect. When compared to the traditional heterotrophic microorganisms that require additional carbohydrates for cofactor regeneration, photoautotrophic PNSB-based biocatalyst might avoid competition with food industry and significantly reduce the operating cost.

We demonstrated light-powered biotransformation for VA and *p*HBA productions using the endogenous CoA-dependent non-β-oxidation route of *R. palustris* together with NADH-dependent ADH2 from *S. cerevisiae*. Both FA and *p*CA were efficiently converted to the corresponding “C_n-2_” alcohols, with ~ 100% conversion under light-anaerobic conditions. The main ways to VA synthesis are via direct extraction from host plants, and chemosynthesis from vanillin (e.g. Pd/C, Pt/C, and Au/C) [33]. Recently, whole-cell bioconversion of 1 g/L 3,4-dihydroxybenzyl alcohol to 499.36 mg/L VA with 45.1% conversion was obtained by *E. coli* [34], and 3.89 g/L VA was produced from glucose and glycerol by *E. coli* in fed-batch fermentation [35]. Traditional *p*HBA biosynthesis is either extracted from *Gastrodia elata* or obtained as the intermediate metabolite [36]. We previously achieved the biotransformation of 5 mM _L_-tyrosine to 581 mg/L *p*HBA with 93.6% conversion using *E.coli* whole-cell catalysis [37]. When compared to previous studies, we have reached better titers of VA (7.7 g/L) and *p*HBA (6.21 g/L), suggesting that *R. palustris* is an appealing and feasible host for light-powered lignocellulose valorization. In the future, it will be possible to engineer *R. palustris* to synthesize diverse aromatic compounds such as vanillin, vanillylamine [38], protocatechuic acid and gallic acid [12] in a similar manner. In addition, we also developed a biocatalytic system for pinene synthesis from isoprenol utilizing IUP. It is reported that an engineered whole-cell catalytic system of *E. coli* improved pinene titer to 166.5 mg/L using sucrose as the carbon source and the substrate, and 0.97 g/L pinene was produced from glucose by an engineered *E. coli* under fed-batch fermentation conditions [39]. In this study, 1.88 g/L pinene with 92% conversion was achieved, which was much higher than the titers obtained by traditional biocatalysis and metabolic engineering efforts [39, 40]. For the future production of other terpenes such as sesquiterpenes, it will require a proper balance of DMAPP and IPP levels by adjusting Idi expression or simply use a fixed ratio of prenol and isoprenol without introducing Idi-mediated isomerization to achieve the theoretical maximum.

To further improve light-powered biotransformation, we also engineered the carotenoid biosynthetic pathway of *R. palustris* to weaken the light shading effect and improve the transmission of light through the cell culture. Our results confirmed that the engineered strains with lower light absorption could accelerate the conversion process. It was reported that disruption of *puc* operon [41] or overexpression of *pufQ*, a regulatory gene to *puf* operon [42], could decrease the pigment contents with low light absorption, which might be conducive to ATP and NADH synthesis. Due to photophosphorylation coupling with electron transport during photosynthesis, slight overexpression of *cycA* encoding for cytochrome *c*_2_, an important electron carrier in electron transport chain, was found to improve ATP and NADH synthesis [43]. In addition, transposon mutagenesis screening has identified several mutants with reduced pigments [44, 45]. These engineering strategies might be similarly implemented in our light-powered biotransformation to further improve the strain performance in the future work.

## Conclusion

In summary, we have developed PNSB-based biocatalysts for light-driven cofactor regeneration to power the whole-cell catalysis. Using a highly active expression system, blocking the side-reactions, controlling the feeding strategy and attenuating the light shading effect, both lignocellulose derivatives of FA and *p*CA were, respectively, transformed into 7.7 g/L VA and 6.21 g/L *p*HBA with ~ 100% conversion under light-anaerobic conditions. Besides, 1.88 g/L pinene with 92% conversion was achieved from isoprenol using IUP. Compared to traditional whole-cell biocatalysis, the autotrophic *R. palustris* as a biocatalyst with cofactor regeneration powered by light instead of carbohydrates, could significantly reduce the cost for future industrial development.

## Materials and methods

### Strains and cultivation

*R. palustris* CGA009 was employed as the base strain to engineer the chassis cell for biotransformation, and the engineered strains with plasmids were maintained with antibiotics (10 μg/mL kanamycin, and 12 μg/mL gentamycin) when needed. For chemoheterotrophic growth, *R. palustris* was aerobically cultivated in 10 mL Sistrom’s mineral medium (MedS) [46] on a shaker (ZQLY-180, Shanghai Zhichu Instrument Co., Ltd, China) at 35 °C and 250 rpm. For photoautotrophic growth, 10 mL modified Sistrom’s mineral medium (MedC) with succinate replaced by 10 mM NaHCO_3_ and 1 mM Na_2_S_2_O_3_ was used for cultivating *R. palustris,* and serum bottles were pressurized with 50 kPa of 80% H_2_/20% CO_2_ sealed by butyl rubber stoppers [47] and incubated at 35 °C under LED bulbs providing 5000 lx irradiance. *E. coli* S17-1 was applied as the competent cell for plasmid construction and cultivated in Luria–Bertani (LB) medium supplemented with antibiotics (50 μg/mL kanamycin, and 12 μg/mL gentamycin) when needed. All strains used in this study are listed in Additional file 1: Table S1. All the chemicals used in the media were purchased from Sangon Biotech (Shanghai, China) or otherwise stated.

### Plasmids construction

PCR amplification of all genes was performed with High Fidelity Phusion DNA polymerase or Taq polymerase from New England Biolab (Ipswich, MA, USA). All restriction enzymes and T4 DNA ligase were obtained from New England Biolab (Ipswich, MA, USA). The rrnB T1 terminator was synthesized by GenScript (Nanjing, China) cut with *Xho*I and *Sal*I, then cloned into pBdRSf [48] via *Xho*I to give pBRT confirmed by sequencing. The untranslated regions of approximately 500 bp upstream from *puc*, *puf*, *bch* and *crt* operons were amplified from the genomic DNA of *R. palustris* CGA009 with primers Ppuc-F/Ppuc-R, Ppuf-F/Ppuf-R, PbchP-F/PbchP-R, and PcrtE-F/PcrtE-R, and then cloned into pBRT between *Eco*RI and *Kpn*I sites to obtain pBRPpuc, pBRPpuf, pBRPbchP, and pBRPcrtE, respectively. The P_T334-6_ promoter [18] was synthesized by GenScript (Nanjing, China) then cloned into pBRT between *Eco*RI and *Kpn*I sites to yield pBRPt334-6, and the DNA fragments containing the P_T334-6_ promoter and the oxygen-regulatory protein binding site of P_*puc*_ from *R. sphaeroides* attained by PCR with primers Pt334O-F/Pt334O-R was digested with *Eco*RI and *Kpn*I and then cloned into pBRT to produce pBRPt334O. Besides, P_*lac*_ and P_*tac*_ promoters amplified from the vector pBBR1MCS-2 and pBBR-tacGFP [29] with primers Plac-F/Plac-R and Ptac-F/Ptac-R were, respectively, cloned into pBRT digested by *Eco*RI and *Kpn*I to produce pBRPlac and pBRPtac. Finally, the *egfp* fragment was cloned into these plasmids via *Bam*HI and *Xho*I to give pBRpuc-eGFP, pBRpuf-eGFP, pBRbchP-eGFP, pBRcrtE-eGFP, pBRt334-6-eGFP, pBRt334O-eGFP, pBRlac-eGFP, and pBRtac-eGFP, respectively.

To replace the kanamycin resistance marker of pBRPt334O to gentamycin resistance, the DNA fragment of gentamycin resistance was amplified from plasmid pZJD29c with primers Gen-F/Gen-R, and then circular PCR was used to create pGenPt334O with gentamycin resistance. To obtain pBRT334O-CouBA, CoA ligase gene (*couB*) [26] and enoyl-CoA hydratase/aldolase gene (*couA*) [26] were, respectively, amplified from *R. palustris* genome using primers couB-F/couB-R and couA-F/couA-R, and then cloned together into pBRPt334O between *Bam*HI and *Xho*I sites. To acquire pGenT334O-ADH2, alcohol dehydrogenase 2 (ADH2 encoded by *adh2*) gene from *S. cerevisiae* [49] amplified with primers ADH2-F/ADH2-R was cloned into pGenPt334O between *Bam*HI and *Xho*I sites. The fragments of hydroxyethylthiazole kinase (ThiM) gene from *E. coli* amplified with primers ThiM-F/ThiM-R, isopentenyl phosphate kinase (IPK) gene from *A. thaliana* amplified with primers IPK-F/IPK-R, and isopentenyl pyrophosphate isomerase (Idi) gene from *E. coli* amplified with primers Idi-F/Idi-R were cloned together into pBRPt334O digested with *Bam*HI and *Xho*I to give pBRT334O-ThiM-IPK-Idi. The fragments of GPPS-PS fusion amplified from pBBR-αGppsPs [29] with primers GPPSps-F1/GPPSps-R1 and GPPSps-F2/GPPSps-R2 were cloned into pGenPt334O digested with *Bam*HI and *Xho*I to give pGenT334O-GPPSps. All oligonucleotides synthesized from GenScript (Nanjing, China) and plasmids used in this study are listed in Additional file 1: Table S2 and S3, respectively.

### Di-parental conjugation

Conjugation mating was implemented for transforming plasmids into *R. palustris*. *E. coli* S17-1 bearing the desired plasmids and *R. palustris* were employed as donor strain and receptor strain, respectively. After *R. palustris* was aerobically cultivated in 10 mL MedS at 35 °C for 24 h and *E. coli* S17-1 was cultivated in 5 mL LB at 37 °C for overnight, the cells were harvested at 7000 rpm for 10 min and washed once with fresh MedS, and resuspended with 1 mL MedS. Then *E. coli* and *R. palustris* were mixed as a ratio of 3:10 (V/V), and the mixture was transferred on a MedS agar plate incubating at 35 °C for 24 h. The cultures were harvested, washed once with fresh MedS, resuspended with 800 μL MedS, and spread on a MedS agar plate with appropriate antibiotics. Colonies typically appeared after 2–3 days at 35 °C.

### Genome editing in* R. palustris*

The suicide plasmid pZJD29c [50] mediated homologous recombination was used for genome editing in *R. palustris*. To attain pZJ-ΔALDH1 for *RPA1206* deletion, the upstream and downstream fragments of the *aldh1* gene were amplified from CGA009 genome with respective primers ALDH1-F1/ALDH1-OE-R1 and ALDH1-OE-F2/ALDH1-R2 assembled by overlapping PCR, then cloned into the *Sac*I and *Kpn*I sites on pZJD29c. The similar way was used to generate pZJ-ΔALDH2 for *RPA1687* deletion and pZJ-ΔALDH3 for *RPA1725* deletion. To implement the promoter replacement, the upstream and downstream fragments of the *ispA* promoter amplified from *R. palustris* CGA009 genome with respective primers ispA-uF/ispA-OE-uR and ispA-OE-dF/ispA-dR and the fragment of P_*lac*_ promoter amplified from pBBR1MCS-2 with primers Plac-OE-iF/Plac-OE-iR were assembled by overlapping PCR and cloned into pZJD29c cut with *Xba*I and *Kpn*I to generate pZJ-Plac-ispA. Based on the similar method, pZJ-Plac-crtE was attained using primers crtE-uF/crtE-OE-uR, Plac-OE-cF/Plac-OE-cR, and crtE-OE-dF/crtE-dR. Single colonies harboring pZJD29c-derived plasmids were picked and cultivated in 1 mL MedS for overnight, and then screened on MedS plate containing 10% sucrose until colonies appeared. Positive mutants were confirmed by diagnostic PCR and DNA sequencing.

### Fluorescence intensity assays

To measure eGFP fluorescence, *R. palustris* recombinants were aerobically cultivated in 5 mL MedS at 35 °C and 250 rpm until OD_630_ (optical density at 630 nm) reached approximately 0.6, then the culture was cultivated at 30 °C under aerobic conditions at 250 rpm or light-anaerobic conditions for 2 days. For incubation at a low oxygen tension, the culture was cultivated at 30 °C under semi-aerobic conditions at 50 rpm by tightening the lid [19]. OD_630_ values and fluorescence intensities were analyzed with a microplate reader, Synergy H1 (BioTek, VT, USA). The excitation and emission of eGFP were set at 485 nm and 510 nm respectively, and the fluorescence intensities were normalized to OD_630_.

### RNA preparation and real-time qPCR

Total RNA of *R. palustris* was extracted with the Bacterial RNA Kit (Omega Bio-Tek, GA, USA) based on the protocol and treated with DNase I to remove the genomic DNA. Then, cDNA synthesis was conducted with M-MuLV First Strand cDNA Synthesis Kit (Sangon Biotech, China) with Oligo(dT) as primers according to the instruction. Real-time qPCR was carried out using Perfectstart SYBR Green qPCR Master Mix kit (Omega Bio-Tek, GA, USA) with a thermal cycler (Agilent AriaMx, Agilent, USA) and the corresponding primers are presented in Additional file 1: Table S2. The expression levels of genes were analyzed by relative quantification-*∆∆Ct* method in which *Ct* values were determined from house-keeping gene (16S rRNA) and target genes in test strains.

### Biocatalysis procedures

The recombinant strains of *R. palustris* were aerobically incubated in 5 mL MedS at 35 °C and 250 rpm for overnight. Then the fresh culture was transferred into 250 mL shake flasks containing 100 mL MedC and aerobically grown at 35 °C and 250 rpm until OD_630_ attained approximately 0.6. Afterwards, the culture was transferred into serum bottles incubated at 30 °C under photoautotrophic conditions facilitating for protein expression.

After reaching an OD_630_ of 1.8–2.0, the cells were harvested by centrifugation at 7000 rpm, 4 °C for 10 min and washed once with ice-cold water and once with KP buffer (200 mM, K_2_HPO_4_·3H_2_O 42.90 g/L, KH_2_PO_4_ 1.63 g/L, pH 7.0), then resuspended in KP buffer to a cell density of 50 g-cdw/L. For biotransformation, 2 mL reaction mixture in injection syringes generally comprised of 10 g-cdw/L cell suspension, a certain amount of substrate, 20 mM NaHCO_3_, 5 mM Na_2_S_2_O_3_ and KP buffer (200 mM, pH 7.0). Besides, 5 mM MgCl_2_ and 1 mM MnCl_2_ were supplemented for pinene synthesis. All biotransformations were carried out on an incubator (ZQZY-75AGN, Shanghai Zhichu Instrument Co., Ltd, China) at 30 °C under dark- and light-anaerobic conditions.

### Analytical methods

Ferulic acid,* p*-coumaric acid, *p*-hydroxybenzyl alcohol, *p*-hydroxybenzoic acid, vanillyl alcohol, and vanillic acid in the aqueous phase were analyzed by a high-performance liquid chromatography (HPLC, Shimadzu LC-20A, Japan) equipped with a photodiode array detector and a reversed-phase column (C18, 150 mm × 4.6 mm × 5 μm) under 40 °C. Mobile phase: 70% ultrapure H_2_O with 0.1% trifluoroacetic acid and 30% acetonitrile. Flow rate: 1 mL/min. Detection wavelength: 210 nm. The reaction mixture from the aqueous phase was centrifuged at 13000 rpm for 10 min at room temperature, and then the supernatant was collected, diluted 10–50 times with ddH_2_O, and filtered before HPLC analysis. Isoprenol and pinene in the organic phase were measured by a gas chromatography (GC, Shimadzu GC-2030, Japan) equipped with a flame ionization detector and a Rtx-5 column (30 m × 0.25 mm × 0.25 μm). Nitrogen was used as a carrier with a flow rate of 1.0 mL/min. The column temperature was first kept at 80 °C for 2 min, then increased to 190 °C at a rate of 5 °C/min, and finally increased to 300 °C by 20 °C/min. 200 μL upper dodecane phase from the reaction mixture was collected and centrifuged at 13,000 rpm for 10 min to remove all cellular particles, and then was diluted 1–20 times with ethyl acetate for GC analysis. The standards used for HPLC analysis were dissolved in ethanol and prepared with 0.1, 0.2, 0.4, 0.6, 0.8, 1.0 mM for plotting the standard curve. The standards used for GC analysis were dissolved in ethyl acetate and prepared with 0.1, 0.2, 0.4, 0.6, 0.8, 1.0 mM for plotting the standard curve. The product and substrate levels were calculated accordingly. All the authentic standards used in GC and HPLC analysis were obtained from Aladdin Biotech (Shanghai, China). The statistical significance of data (*p* value) was analyzed with GraphPad Prism 8 using a *t* test.

### Supplementary Information

**Additional file 1: Table S1.** Strains used in this study. **Table S2.** Oligonucleotides used in this study. **Table S3.** Plasmids used in this study. **Figure S1.** *R. palustris* contained two plasmids for synthesis of vanillyl alcohol (VA) or *p*-hydroxybenzyl alcohol (*p*HBA). **Figure S2.** Effects of *aldh* deletions on *R. palustris*. **Figure S3.** Effects of the decreases in *ispA* and *crtE* expressions on *R. palustris*. **Figure S4.** Synthesis of *p*HBA from *p*CA using whole-cell biocatalysis. **Figure S5.** The HPLC result for the production of *p*HBA from *p*CA. **Figure S6.** *R. palustris* contained two plasmids for pinene synthesis from isoprenol. **Figure S7.** The GC result for the synthesis of pinene from isoprenol.

## Data Availability

All data generated or analyzed during this study are included in this published article and its Additional files.
