# Supplementary material for: Multidimensional optimization for accelerating light-powered biocatalysis in Rhodopseudomonas palustris
Source: Biotechnol Biofuels Bioprod. 2023 Oct 27;16:160. doi: 10.1186/s13068-023-02410-3 (PMC10612212; doi:10.1186/s13068-023-02410-3)
Supplement: Supplementary file 1 — Additional file 1: Table S1. Strains used in this study. Table S2. Oligonucleotides used in this study. Table S3. Plasmids used in this study. Figure S1. R. palustris contained two plasmids for synthesis of vanillyl alcohol (VA) or p-hydroxybenzyl alcohol (pHBA). Figure S2. Effects of aldh deletions on R. palustris. Figure S3. Effects of the decreases in ispA and crtE expressions on R. palustris. Figure S4. Synthesis of pHBA from pCA using whole-cell biocatalysis. Figure S5. The HPLC result for the production of pHBA from pCA. Figure S6. R. palustris contained two plasmids for pinene synthesis from isoprenol. Figure S7. The GC result for the synthesis of pinene from isoprenol. [file 13068_2023_2410_MOESM1_ESM.docx]

**Supporting Information**

**Multidimensional optimization for accelerating light-powered biocatalysis in *Rhodopseudomonas palustris***

Yang Zhang^1,2^, Wenchang Meng,^1^ Yuting He,^1^ Yuhui Chen,^1^ Mingyu Shao,^1^ and Jifeng Yuan^1,2^*

^1^State Key Laboratory of Cellular Stress Biology, School of Life Sciences, Faculty of Medicine and Life Sciences, Xiamen University, Fujian 361102, China

^2^Shenzhen Research Institute of Xiamen University, Shenzhen 518057, China

*Corresponding author email: jfyuan@xmu.edu.cn

**Supplementary Table S1. Strains used in this study.**

| Strain | Description | Source |
| --- | --- | --- |
| *E. coli* S17-1 | *thi* *pro* *hsdR* *hsdM*^+^ *recA*::(RP4-2-Tc::Mu-Km::Tn7) λpir, Sm^r^ | ^1^ |
| *R. palustris* CGA009 | Wild type | ^2^ |
| YC1 | CGA009 with *RPA1206* gene deletion | This study |
| YC2 | CGA009 with *RPA1206*, *RPA1687* and *RPA1725* genes deletion | This study |
| YC3 | YC2 with *ispA* promoter replaced by P*_lac_* | This study |
| YC4 | YC2 with both *ispA* and *crtE* promoters replaced by P*_lac_* | This study |
| YC5 | CGA009 with *ispA* promoter replaced by P*_lac_* | This study |
| YC6 | CGA009 with both *ispA* and *crtE* promoters replaced by P*_lac_* | This study |
| RVA1 | CGA009 harboring plasmids pBRT334O-CouBA and pGenT334O-ADH2 | This study |
| RVA2 | YC1 harboring plasmids pBRT334O-CouBA and pGenT334O-ADH2 | This study |
| RVA3/RHBA1 | YC2 harboring plasmids pBRT334O-CouA and pGenT334O-ADH2 | This study |
| RVA4/RHBA2 | YC4 harboring plasmids pBRT334O-CouA and pGenT334O-ADH2 | This study |
| PE1 | CGA009 harboring plasmids pBRT334O-ThiM-IPK-Idi and pGenT334O-GPPSps | This study |
| PE2 | YC5 harboring plasmids pBRT334O-ThiM-IPK-Idi and pGenT334O-GPPSps | This study |
| PE3 | YC6 harboring plasmids pBRT334O-ThiM-IPK-Idi and pGenT334O-GPPSps | This study |

**Supplementary Table S2. Oligonucleotides used in this study.**

| Ppuc-F | CG*GAATTC*TGGACGATGGTCAAATCCG |
| --- | --- |
| Ppuc-R | TT*GGTACC*TCCTCCTGAGAGACTTACG |
| Ppuf-F | CG*GAATTC*AGGGTTCTTCCGGATAGT |
| Ppuf-R | TT*GGTACC*TCACCTCCTAGTGATGG |
| PbchP-F | CG*GAATTC*TGCTCGATCGCTGGCTCG |
| PbchP-R | TT*GGTACC*CGGCTCCGTCTCCTTCCG |
| PcrtE-F | CG*GAATTC*TAACGCTTCGCTTCCGCA |
| PcrtE-R | TT*GGTACC*ACACTCCCGCGCTTCAGG |
| Pt334O-F | CG*GAATTC*TGTCTCTCTCCTGCCGTCC |
| Pt334O-R | TT*GGTACC*GGCAAAATTGTCCCTTTTCAAGTGCCTCCTTCAGATGCAA |
| Plac-F | CG*GAATTC*CGCAACGCAATTAATGTG |
| Plac-R | TT*GGTACC*GCTGTTTCCTGTGTGAAA |
| Ptac-F | CG*GAATTC*CGGTTCTGGCAAATATTC |
| Ptac-R | TT*GGTACC*TCCTGTGTGAAATTGTTA |
| Gen-F | GACAGGATGAGGATCGTTTCGCATGTTACGCAGCAGCAACG |
| Gen-R | GTCATTTCGAACCCCAGAGTCCCGCTTAGGTGGCGGTACTTGG |
| ThiM-F | TT*CGTCTC*GGATCCGAGGAGGTATATTATGCAAGTCGACCTGCTGG |
| ThiM-R | TT*CGTCTC*ACTCCTCATGCCTGCACCTCCTGC |
| IPK-F | TT*CGTCTC*AGGAGGTATATTATGGAGCTGAATATTTCCG |
| IPK-R | TT*CGTCTC*ATCCTCTACTTTGAGAATCTGATG |
| Idi-F | TT*CGTCTC*AAGGAGGTATATTATGCAAACGGAACACGTC |
| Idi-R | TT*CGTCTC*CTCGAGTTATTTAAGCTGGGTAAATGC |
| GPPSps-F1 | TT*GGTCTC*GGATCCGAGCAGAGGAGAACTAGTAT |
| GPPSps-R1 | TT*GGTCTC*ACGACACCTTGCCGTTTTCATAATA |
| GPPSps-F2 | TT*GGTCTC*TGTCGTGCGGCCATCGGATC |
| GPPSps-R2 | TT*GGTCTC*CTCGAGTCAGAGGGGGACCGACTCG |
| couA-F | TT*CGTCTC*AAGCTTAGGAGGAATAAAGTGCTCACAGGAAACGCTCA |
| couA-R | TT*CGTCTC*CTCGAGTCAGCTCGGCCGAACCTTGG |
| couB-F | CG*GGATCC*AGGAGGAATAAAGTGATGGACGCCATGACCGA |
| couB-R | AGAG*AAGCTT*TCACTCCAGCGCAATCACAT |
| ADH2-F | CG*GGATCC*AGGAGGAATAAAATGTCTATTCCAGAAACTCA |
| ADH2-R | AGAGA*CTCGAG*TTATTTAGAAGTGTCAACAACG |
| ispA-uF | TT*GGTACC*TCTGGAACGCTTCACCGA |
| ispA-OE-uR | ATTAATTGCGTTGCGGGAGCACTTCCGAATGGC |
| Plac-OE-iF | ATTCGGAAGTGCTCCCGCAACGCAATTAATGTG |
| Plac-OE-iR | CGGCCGAATTTTATCTGCTGTTTCCTGTGTGAAA |
| ispA-OE-dF | ACACAGGAAACAGCAGATAAAATTCGGCCGGACC |
| ispA-dR | GC*TCTAGA*CTCCTTGGCGGACAACTGCG |
| crtE-uF | GC*TCTAGA*ACCGCAATAGGTCGCATAGC |
| crtE-OE-uR | TTAATTGCGTTGCGCAGGCACAAGTGTCAGTTTA |
| Plac-OE-cF | CTGACACTTGTGCCTGCGCAACGCAATTAATGTG |
| Plac-OE-cR | CATGGCCACACTCCCGCTGTTTCCTGTGTGAAA |
| crtE-OE-dF | ACACAGGAAACAGCGGGAGTGTGGCCATGGACG |
| crtE-dR | TT*GGTACC*GGCAACCTGATAGGCTTCG |
| ALDH1-F1 | AGAG*GAGCTC*ATCTTCACCTGGCGTTCCT |
| ALDH1-OE-R1 | CTCATCACAACGATGGCCCACTCGGCTTCTTCTA |
| ALDH1-OE-F2 | AGAAGCCGAGTGGGCCATCGTTGTGATGAGGTTCA |
| ALDH1-R2 | TT*GGTACC*TGAGAATGACGACACTGCCG |
| ALDH2-F1 | GC*TCTAGA*GCCATCGTGCTGGGTGAGT |
| ALDH2-OE-R1 | TTTCATCGCATGTTCGGCGGCTGTCGAAGGTGCTG |
| ALDH2-OE-F2 | ACCTTCGACAGCCGCCGAACATGCGATGAAAGCTG |
| ALDH2-R2 | TT*GGTACC*GGGTGCCAACGAAACCAAGC |
| ALDH3-F1 | GC*TCTAGA*CTGGCGGAAGTCGGACGGGTG |
| ALDH3-OE-R1 | ATCGTCAAGAGATCACAGGCTTTGCAGGATGGCGGT |
| ALDH3-OE-F2 | CATCCTGCAAAGCCTGTGATCTCTTGACGATGAGCG |
| ALDH3-R2 | GG*GGTACC*TGTCGATACCGACATAGGTG |
| 16Sr-qPCR-F | GTCATCCCCACCTTCCTCGC |
| 16Sr-qPCR-R | ATGGCTGTCGTCAGCTCGTG |
| ispA-qPCR-F | GACGAAGGTGGTCTTGCCGA |
| ispA-qPCR-R | GTGAAGCGTTCCAGATTGCC |
| crtE-qPCR-F | GAAATCAATCTCGCCCATTA |
| crtE-qPCR-R | GATCTTCTCACCGACCATCC |

**Supplementary Table S3. Plasmids used in this study.**

| Plasmid | Description | Source |
| --- | --- | --- |
| pBBR1MCS-2 | Broad-host-range vector, Km^r^ | ^3^ |
| pBBR-αGppsPs | pBBR1MCS-2 derivative containing GPPS-PS fusion | ^4^ |
| pZJD29c | Mobilizable suicide vector, Gent^r^ | ^5^ |
| pBdRSf | pBBR1MCS-2 derivative removing MCS and P*_lac_* | ^6^ |
| pBRT | pBRT derivative containing the terminator rrnB T1 | This study |
| pBRPpuc | pBRT derivative carrying the promoter of *puc* operon | This study |
| pBRPpuf | pBRT derivative carrying the promoter of *puf* operon | This study |
| pBRPbchP | pBRT derivative carrying the promoter of *pgk* gene | This study |
| pBRPcrtE | pBRT derivative carrying the promoter of *eno* gene | This study |
| pBRPlac | pBRT derivative carrying the promoter of *lac* operon | This study |
| pBRPtac | pBRT derivative carrying the promoter P*_tac_* | This study |
| pBRPt334-6 | pBRT derivative carrying the promoter P_T334-6_ of *Rhodobacter sphaeroides* | This study |
| pBRPt334O | pBRT derivative carrying the promoter of P_T334-6_ and the oxygen-regulatory protein binding site of P*_puc_* from *R. sphaeroides* | This study |
| pGenPt334O | pBRPt334O derivative with gentamycin resistance instead of kanamycin resistance | This study |
| pBRpuc-eGFP | pBRPpuc derivative harboring the *eGFP* driven by P*_puc_* | This study |
| pBRpuf-eGFP | pBRPpuf derivative harboring the *eGFP* driven by P*_puf_* | This study |
| pBRbchP-eGFP | pBRPbchP derivative harboring the *eGFP* driven by P*_bchP_* | This study |
| pBRcrtE-eGFP | pBRPcrtE derivative harboring the *eGFP* driven by P*_crtE_* | This study |
| pBRPlac-eGFP | pBRPlac derivative harboring the *eGFP* driven by P*_lac_* | This study |
| pBRPtac-eGFP | pBRPtac derivative harboring the *eGFP* driven by P*_tac_* | This study |
| pBRPt334-6-eGFP | pBRPt334-6 derivative harboring the *eGFP* driven by P_T334-6_ | This study |
| pBRPt334O-eGFP | pBRPt334O derivative harboring the *eGFP* driven by P_T334O_ | This study |
| pBRT334O-CouBA | pBRPt334O derivative containing cascade enzymes CouB-CouA | This study |
| pGenT334O-ADH2 | pGenPt334O derivative containing enzyme ADH2 | This study |
| pBRT334O-ThiM-IPK-Idi | pBRPt334O derivative containing cascade enzymes ThiM-IPK-Idi | This study |
| pGenT334O-GPPSps | pGenPt334O derivative containing fusion enzymes GPPS/PS | This study |
| pZJ-Plac-ispA | pZJD29c derivative containing the flanking regions of *ispA* promoter and P*_lac_* fragment between the flanking regions | This study |
| pZJ-Plac-crtE | pZJD29c derivative containing the flanking regions of *crtE* promoter and P*_lac_* fragment between the flanking regions | This study |
| pZJ-ΔALDH1 | pZJD29c derivative containing the flanking regions of *RPA1206* gene | This study |
| pZJ-ΔALDH2 | pZJD29c derivative containing the flanking regions of *RPA1687* gene | This study |
| pZJ-ΔALDH3 | pZJD29c derivative containing the flanking regions of *RPA1725* gene | This study |


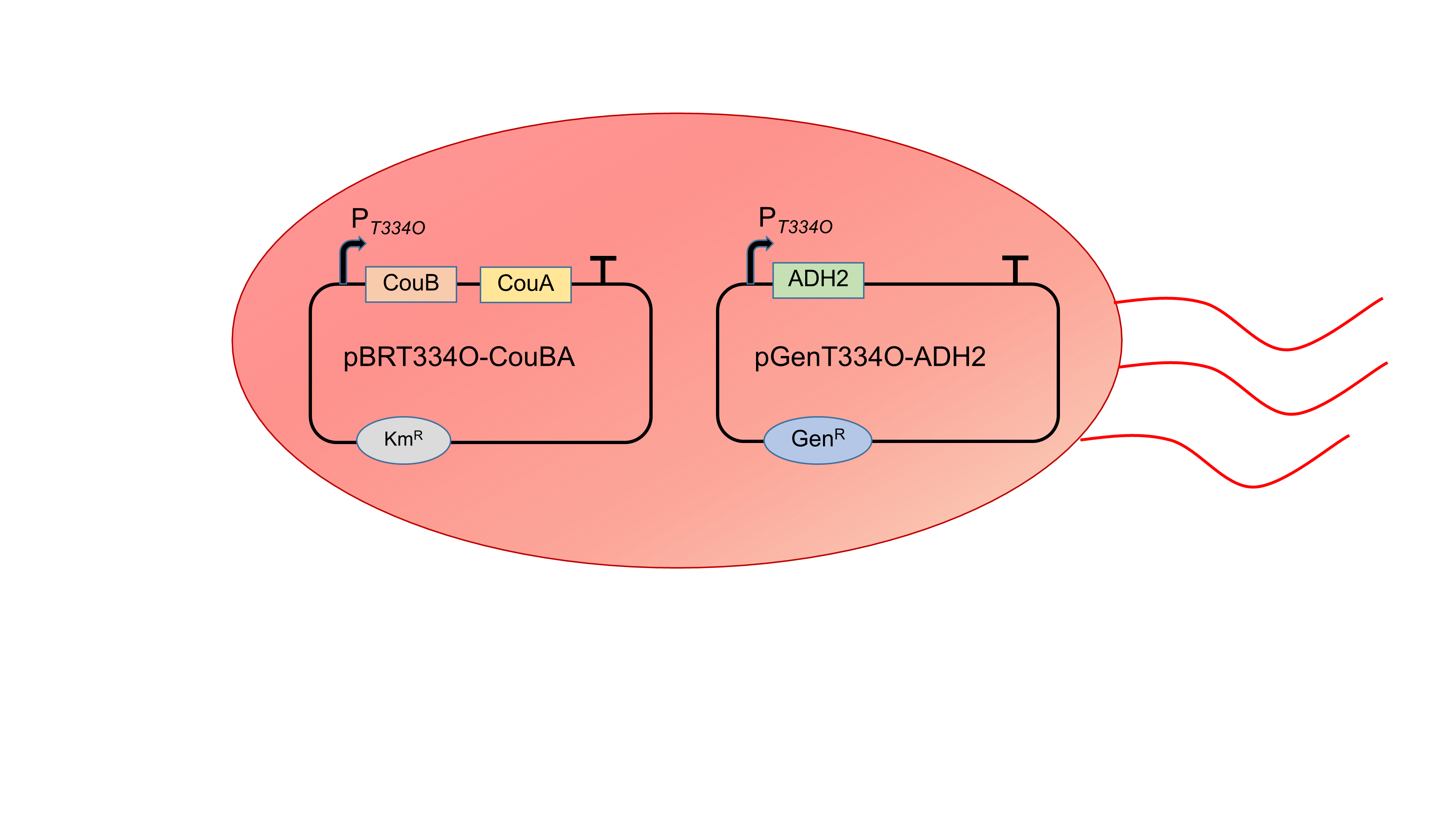


**Figure S1.** *R. palustris* contained two plasmids for synthesis of vanillyl alcohol (VA) or *p*-hydroxybenzyl alcohol (*p*HBA).


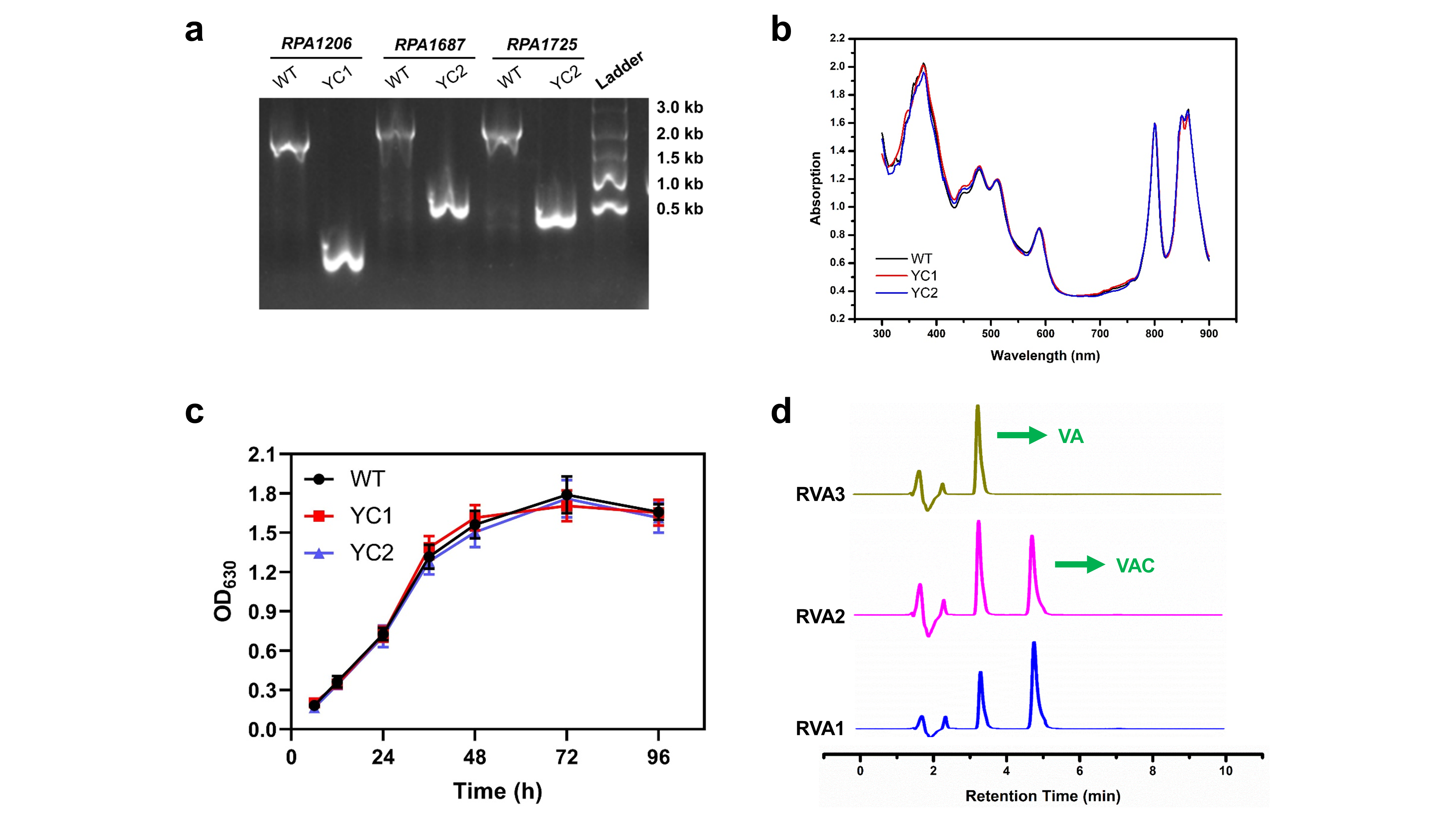


**Figure S2.** Effects of *aldh* deletions on *R. palustris*. a) Agarose gel image for PCR verification of genes knockout. b) The growth profiles of *R. palustris* under light- anaerobic conditions. c) The light absorption of *R. palustris* under light-anaerobic conditions. WT, YC1, and YC2 respectively represent for the base strain, the mutant with *RPA1206* deletion, and the mutant with *RPA1206*, *RPA1687* and *RPA1725* deletions. d) The HPLC results from 5 mM FA using different recombinant strains are shown. Strain RVA1, RVA2, and RVA3 represent for the base strain, YC1, and YC2 respectively containing CouBA-ADH2. Data represent the average of three replicates and error bars represent the standard deviation.


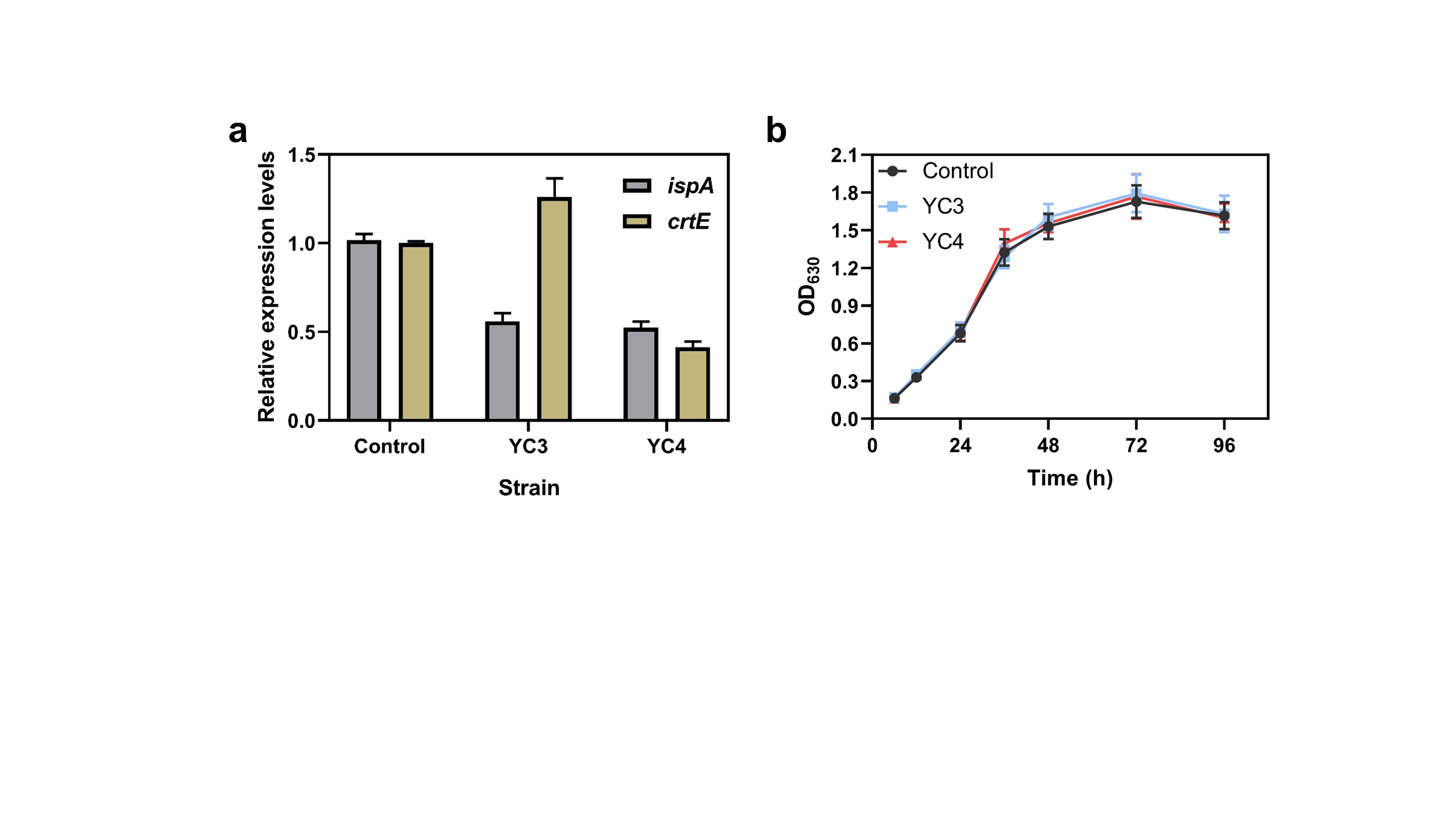


**Figure S3.** Effects of the decreases in *ispA* and *crtE* expressions on *R. palustris*. a) Relative expression levels of *ispA* and *crtE* genes in different strains. b) The growth profiles of *R. palustris* under light-anaerobic conditions. The control represents for strain YC2. Strain YC3 and YC4 respectively represent for decrease in *ispA* and both *ispA* and *crtE* based on strain YC2. Data represent the average of three replicates and error bars represent the standard deviation.


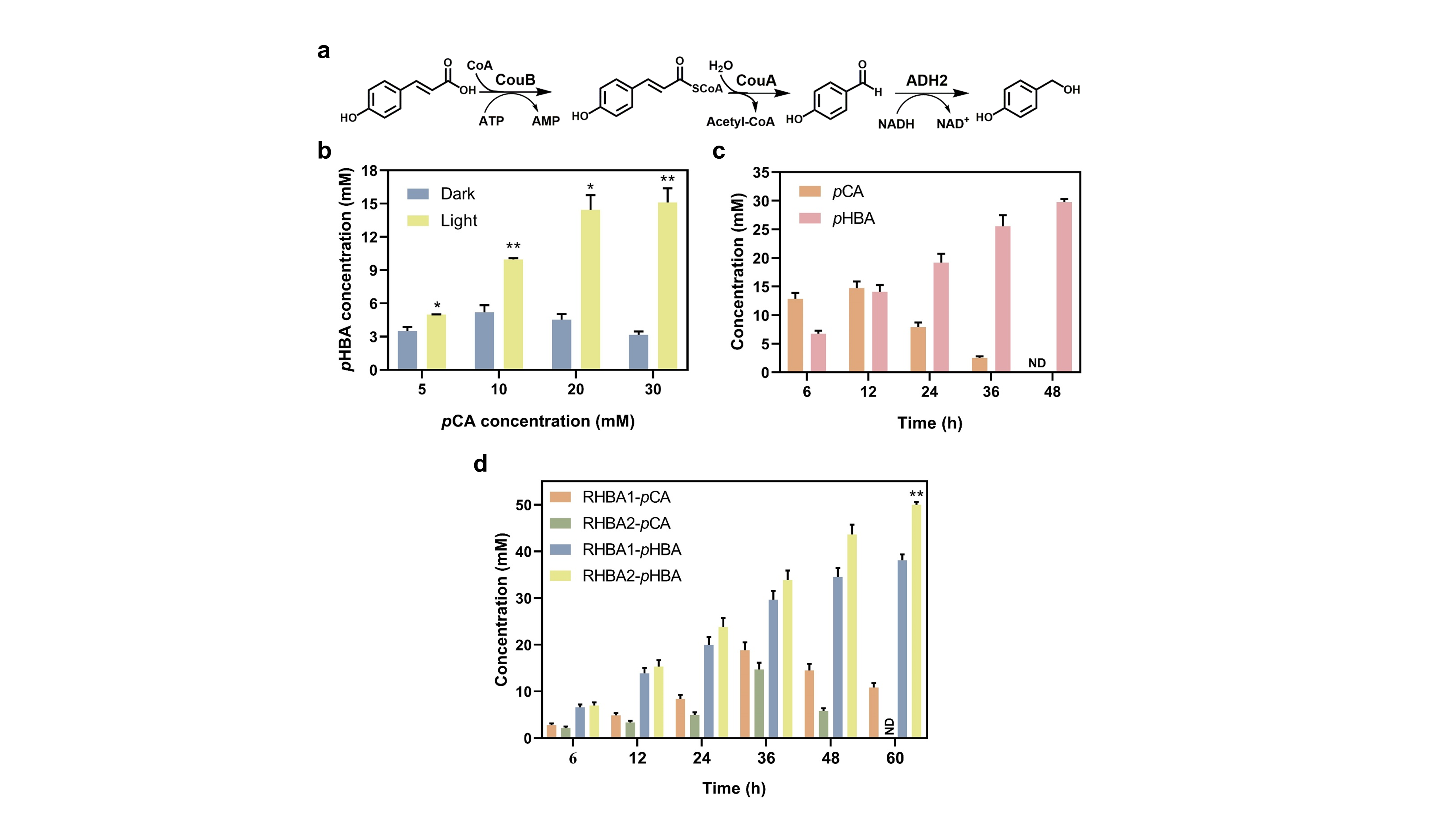


**Figure S4.** Synthesis of *p*HBA from *p*CA using whole-cell biocatalysis. a) Biocatalytic route for converting *p*CA into *p*HBA. b) Bioconversion of *p*CA at different concentrations into *p*HBA using the resting cells of RHBA1 under dark- and light- anaerobic conditions. One asterisk indicates statistically significant results (*p* < 0.05) and two asterisks indicate *p* < 0.005. c) Time course of *p*HBA production from 30 mM *p*CA by strain RHBA1 using the pulse-feeding approach under light-anaerobic conditions. d) *p*HBA synthesis from 50 mM *p*CA using strain RHBA1 and RHBA2 under the pulse-feeding mode. ND, not detectable. All experiments were conducted in triplicate. Data represent the mean and standard deviations.


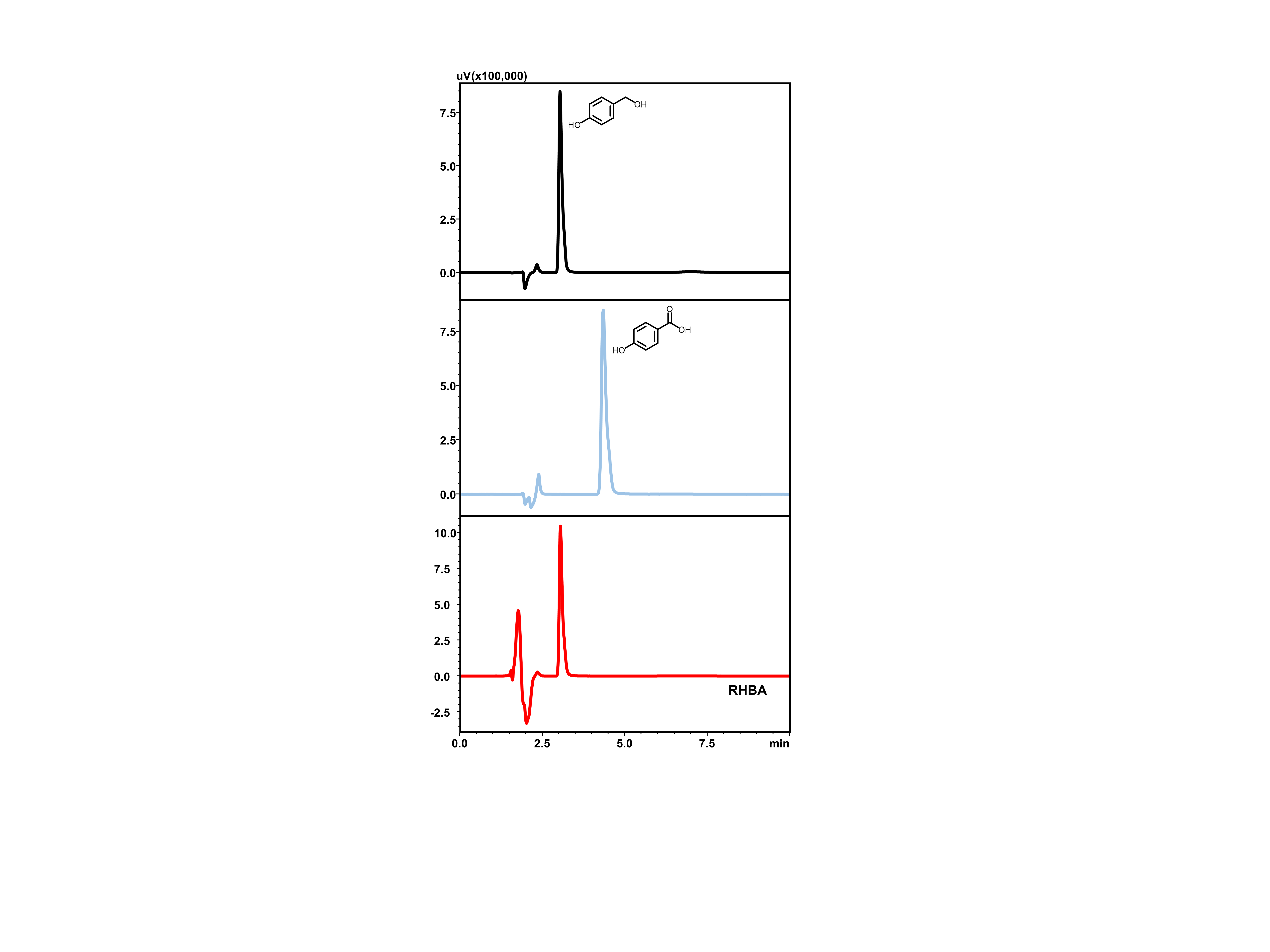


**Figure S5.** The HPLC result for the production of *p*HBA from *p*CA.


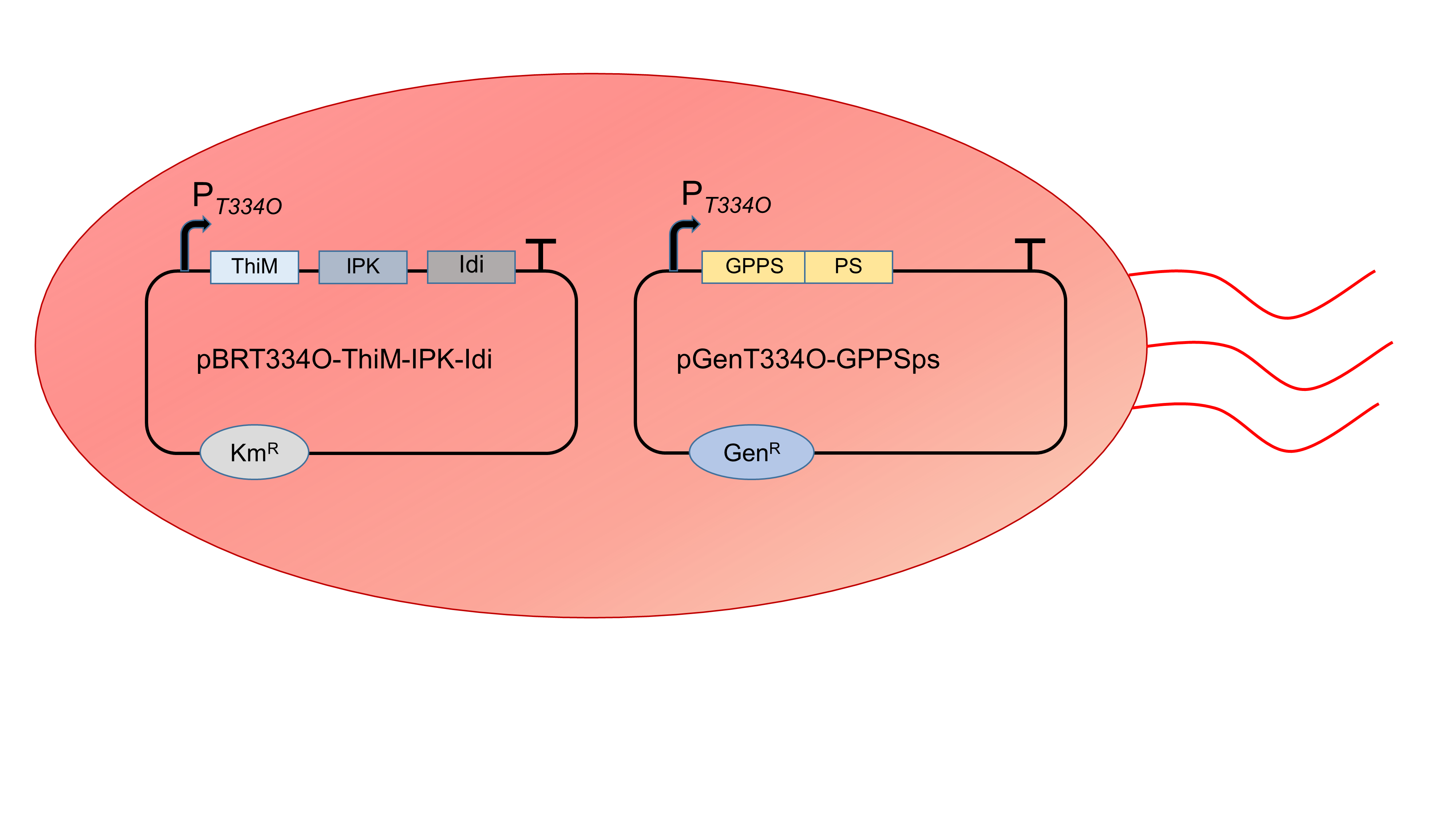


**Figure S6.** *R. palustris* contained two plasmids for pinene synthesis from isoprenol.


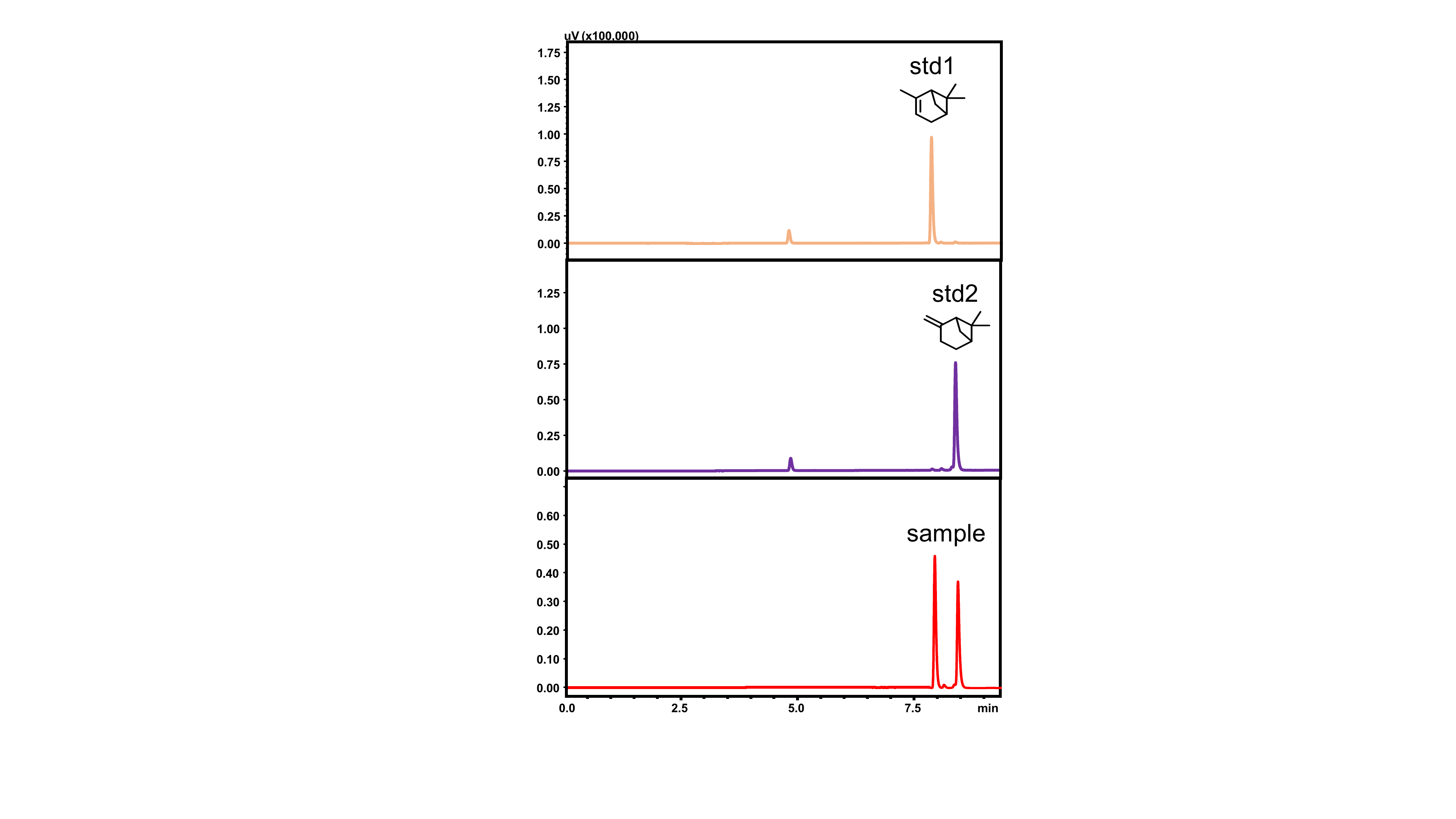


**Figure S7.** The GC result for the synthesis of pinene from isoprenol.

**References**

(1) Penfold, R. J.; Pemberton, J. M. An improved suicide vector for construction of chromosomal insertion mutations in bacteria. *Gene* **1992**, *118* (1), 145-146.

(2) Larimer, F. W.; Chain, P.; Hauser, L.; Lamerdin, J.; Malfatti, S.; Do, L.; Land, M. L.; Pelletier, D. A.; Beatty, J. T.; Lang, A. S.; et al. Complete genome sequence of the metabolically versatile photosynthetic bacterium *Rhodopseudomonas palustris*. *Nat Biotechnol* **2004**, *22* (1), 55-61.

(3) Kovach, M. E.; Elzer, P. H.; Hill, D. S.; Robertson, G. T.; Farris, M. A.; Roop, R. M., 2nd; Peterson, K. M. Four new derivatives of the broad-host-range cloning vector pBBR1MCS, carrying different antibiotic-resistance cassettes. *Gene* **1995**, *166* (1), 175-176.

(4) Wu, X.; Ma, G.; Liu, C.; Qiu, X. Y.; Min, L.; Kuang, J.; Zhu, L. Biosynthesis of pinene in purple non-sulfur photosynthetic bacteria. *Microb Cell Fact* **2021**, *20* (1), 101.

(5) Yano, T.; Sanders, C.; Catalano, J.; Daldal, F. *sacB*-5-Fluoroorotic acid-*pyrE*-based bidirectional selection for integration of unmarked alleles into the chromosome of *Rhodobacter capsulatus*. *Appl Environ Microbiol* **2005**, *71* (6), 3014-3024.

(6) Zhang, Y.; Song, X.; Lai, Y.; Mo, Q.; Yuan, J. High-yielding terpene-based biofuel production in *Rhodobacter capsulatus*. *ACS Synth Biol* **2021**, *10* (6), 1545-1552.
